# Accelerating the experimental responses on cell behaviors: a long-term prediction of cell trajectories using Social Generative Adversarial Network

**DOI:** 10.1038/s41598-020-72605-3

**Published:** 2020-09-24

**Authors:** Maria Colomba Comes, J. Filippi, A. Mencattini, F. Corsi, P. Casti, A. De Ninno, D. Di Giuseppe, M. D’Orazio, L. Ghibelli, F. Mattei, G. Schiavoni, L. Businaro, C. Di Natale, E. Martinelli

**Affiliations:** 1 Department of Electronic Engineering, University of Rome Tor Vergata, Via del Politecnico 1, 00133 Rome, Italy; 2 Interdisciplinary Center for Advanced Studies on Lab-on-Chip and Organ-on-Chip Applications (ICLOC), University of Rome Tor Vergata, 00133 Rome, Italy; 3 Department of Biology, University of Rome Tor Vergata, 00133 Rome, Italy; 4 Institute for Photonics and Nanotechnologies, Italian National Research Council, Via Cineto Romano 42, 00156 Rome, Italia; 5 Department of Oncology and Molecular Medicine, Istituto Superiore Di Sanità, Viale Regina Elena 299, 00161 Rome, Italy

**Keywords:** Biomedical engineering, Electrical and electronic engineering

## Abstract

The incremented uptake provided by time-lapse microscopy in Organ-on-a-Chip (OoC) devices allowed increased attention to the dynamics of the co-cultured systems. However, the amount of information stored in long-time experiments may constitute a serious bottleneck of the experimental pipeline. Forward long-term prediction of cell trajectories may reduce the spatial–temporal burden of video sequences storage. Cell trajectory prediction becomes crucial especially to increase the trustworthiness in software tools designed to conduct a massive analysis of cell behavior under chemical stimuli. To address this task, we transpose here the exploitation of the presence of “social forces” from the human to the cellular level for motion prediction at microscale by adapting the potential of Social Generative Adversarial Network predictors to cell motility. To demonstrate the effectiveness of the approach, we consider here two case studies: one related to PC-3 prostate cancer cells cultured in 2D Petri dishes under control and treated conditions and one related to an OoC experiment of tumor-immune interaction in fibrosarcoma cells. The goodness of the proposed strategy has been verified by successfully comparing the distributions of common descriptors (kinematic descriptors and mean interaction time for the two scenarios respectively) from the trajectories obtained by video analysis and the predicted counterparts.

## Introduction

Thanks to the incremented uptake provided by time-lapse microscopy (TLM) and the use of microfluidic devices to mimic closely the in vivo cellular microenvironments (called Organ-on-a-Chip, OoC), long-term live cell imaging and high-throughput quantification of response dynamics have been recently considered with increased attention^1,2^. In particular, the detection of cell motility, migration patterns, and interactions in multicellular ecosystems have proved to be crucial in OoCs for the modeling and understanding of very complex diseases such as cancer and its related processes of metastasis or mechanisms of cross-talk with the immune system^3–5^.

On the other hand, the huge amount of information stored in long-term time-lapse experiments, involving many cells and events, may constitute one of the most serious bottlenecks of the pipeline. In addition, unwanted effects related to phototoxicity or photobleaching phenomena may also produce misleading consequences and alter the whole comprehension of the experiment^6^. Hence, to increase the trustworthiness in these alternative methods, it is crucial to demonstrate the possibility of performing massive and reliable analysis on large set of living cells interacting and migrating in the reconstituted environment.

In particular, in a multi-cell type environment, most important information relies on the cell movement that acts as a sort of “alarm bell” of underlying biological processes. Understanding the mechanisms of cell movement plays a key role for preventative therapeutic agents to many diseases that cause abnormalities in motility behaviors^7,8^. Indeed, morphogenesis^9^, tissue repair^10^, immune response^11^ but also cancer growth and metastasis^3,4^ involve motility behaviours reflecting the main cell functionalities. Typically, cell motility experiments use time-lapse microscopy imaging techniques that allow to acquire high-frame rate video sequences and reconstitute the position of many cells simultaneously in a label free modality^12^. To date, the image-based system approach has steadily provided great contribution to decipher cellular motion pattern thanks to accurate single-cell segmentation and tracking tools^2,13^. If on one hand, standard quantification of cell motility relies upon the extraction of handcrafted kinematic descriptors^14,15^, on the other hand, deep learning methodologies have recently been developed to discover the “motility style” by learning features directly from cell trajectories^2,16^. Such information may represent a serious drawback due to memory and time required. Therefore, forward prediction of cell trajectories along time becomes a challenging aspect for the reduction of spatial–temporal burden of video sequences storage and analysis.

To the aim, we extended here a neural network approach based on Social Generative Adversarial Network (SGAN) previously described in Gupta et al*.*^17^. The key idea was to transpose the exploitation of the presence of “social forces” from the human to the cellular level for motion prediction at microscale. Actually, cells (as well pedestrians in a realistic scene) can be summarized as interactive agents/objects in motion, animated by chemical (social) forces that make their behavior interpersonal and socially acceptable. Agents can contribute each other to affect their own motion behavior and, as consequence, to move accordingly to avoid collision.

Some approaches have already been tackled in literature to model collective cell behaviour through the use of equations (i.e. diverse versions of Langevin equations)^18,19^ especially stressing that groups of cells may coordinate their motion not as a mere sum of many individually moving cells. Proof of such concept are the presence of diverse cell-roles (leader/follower) in collective migration^20^ or the emergent chemotaxis when cells crawling together^19^.

However, only recently the possibility of predicting cell motility using deep learning has been discussed by Kimmel et al.^16^ and Nishimoto et al*.*^21^ . We can distinguish two categories of systems: image-based^21^ and track-based^16^ methods among which our approach falls. In the work of Nishimoto et al*.*^21^ a Convolutional Neural Network (CNN) predicted cell movement by studying the cell shape change from images. In Kimmel et al*.*^16^ the authors drew up a Recurrent Neural Network (RNN) to predict a certain time steps of cell trajectories in the future including a comparison with a well-known linear baseline method which requires cells to move according to a ballistic motion^22^. The baseline model arose on a priori imposed model of motion, while the RNN architecture accepted cell trajectories of fixed length as input. Moreover, from a biological point of view, drawing cell trajectories as separate and unrelated entities may yield a simplistic model of reality neglecting how each individual cell correlates its own movement with that of its neighbours by means of a shaped social signalling network^23^. To account for the collective behaviour, in Gupta et al*.*^17^, the authors introduced a prediction approach named Social Generative Adversarial Network (SGAN) that was oriented to the short-term prediction of human motion attitude. A social-force collective movement rationale inspired our present work in cell motility and migration studies. Unlike Gupta et al*.*^17^, we endeavoured predictions of long periods with respect to the time-constant involved, by upgrading SGAN using an iterative structure accepting in input ground-truth as well previously predicted cell positions.

Based on the previously mentioned approach, the main scope of this work is to apply the SGAN architecture to predict cell trajectories. A quantitative evaluation of the prediction was executed by computing the average mean squared error (MSE) between the predicted and the corresponding trajectories tracked on real images. Beyond such evaluation, the goodness of the predicted trajectories was assessed through the comparison of the distribution of the statistical descriptors (or interaction parameters) extracted in the predicted and in the corresponding trajectories tracked on real images. The aim was to demonstrate that there was not any statistical difference between the distribution of values and hence that the cell trajectory prediction was effective in providing tracks reliable for the extraction of cell behavior characteristics. Differently from a standard classification procedure, training and testing partition of trajectories were only extracted for the task of SGAN learning procedure as it will be described in the following paragraphs. The obtained result allows not only to prove the practicability of the approach in cell motility studies but also suggests a novel indirect assessment procedure that looks at the usefulness of the extracted trajectories.

To demonstrate the feasibility of the proposed approach, we have firstly evaluated the results obtained with simulated videos where the theoretical trajectories were known. After this preliminary analysis, we considered two distinct biological scenarios in conventional and microfluidic-based cell culture methods. In the first experiment, the SGAN approach transposed the influential “social role” of the clusters of prostate cancer cells on each single cell motion prediction, transferring the ability of the group to “include” or to “exclude” every single cell accordingly.

As a second scenario, we moved from cell motility to cell migration of immune cells toward a tumor cell. Cell migration can be seen as the response to a cell attractant or repellent, acting as the cause of cell motility variation^19^. The “social role” of many immune cells co-targeting towards the same tumor cells, in a sort of competition/completion activity, was here modelled by the SGAN architecture with the aim to predict the trajectories of the immune cells during the task.

Finally, our effort was oriented to accelerate the uptake of the experiments moving from OoCs to Organ in-Silico (OiS) experiments saving information about the biological content inside the OoCs experiments. The OiSs the improvement relies principally on the possibility to conduct massive analysis in parallel, therefore, accelerating the responses on cell behaviour (hence the time-to market) and providing an output ahead of the actual experiment time. Moreover, the invasive effects of phototoxicity and/or photobleaching phenomena may be drastically reduced towards a safer experiment and an even more “in-vivo-like” scenario, with the possibility to acquire with the longer time interval. Thanks to the “social-based-reasoning” of the SGAN architecture, we were able to model the complexity of a multi-population environment and foresee the possibility to investigate even more realistic scenarios towards a deep understanding of human biology.

## Results

### Proposed approach and the experimental set-up

**Figure 1:**
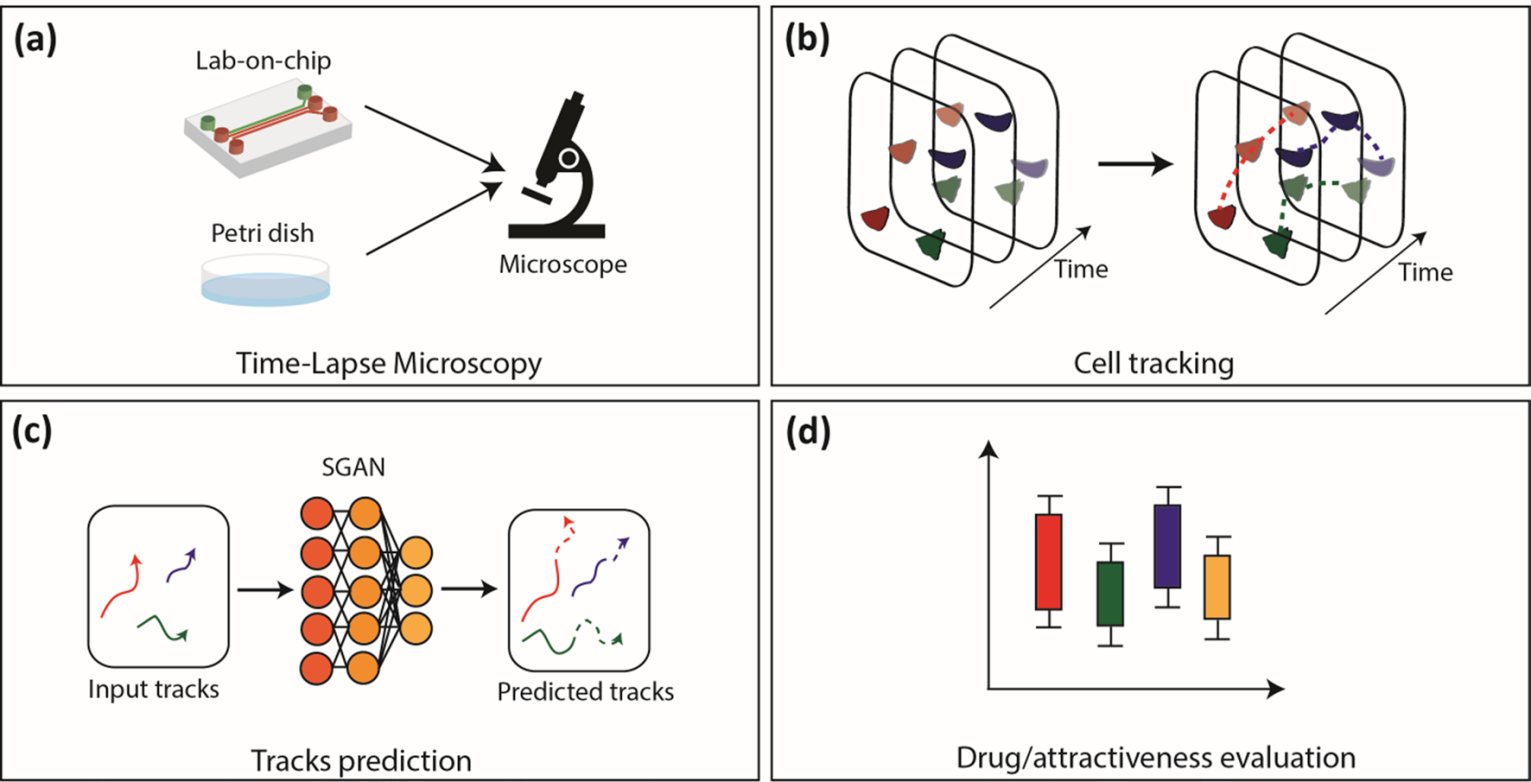
A schematic representation of the proposed method. (**a**) Time-lapse microscopy is used to acquire the video sequence of cells moving in a Petri dish or in an OOC platform. (**b**) Cells are localized and tracked through the video sequence. (**c**) By giving in input a certain number of cell positions for each cell, SGAN predicts future positions for that cell (predicted trajectory) taking into account interactions with neighbouring cells. (**d**) Drug/attractiveness of multiple experimental scenarios are evaluated by extracting features from predicted trajectories.

**Figure 2:**
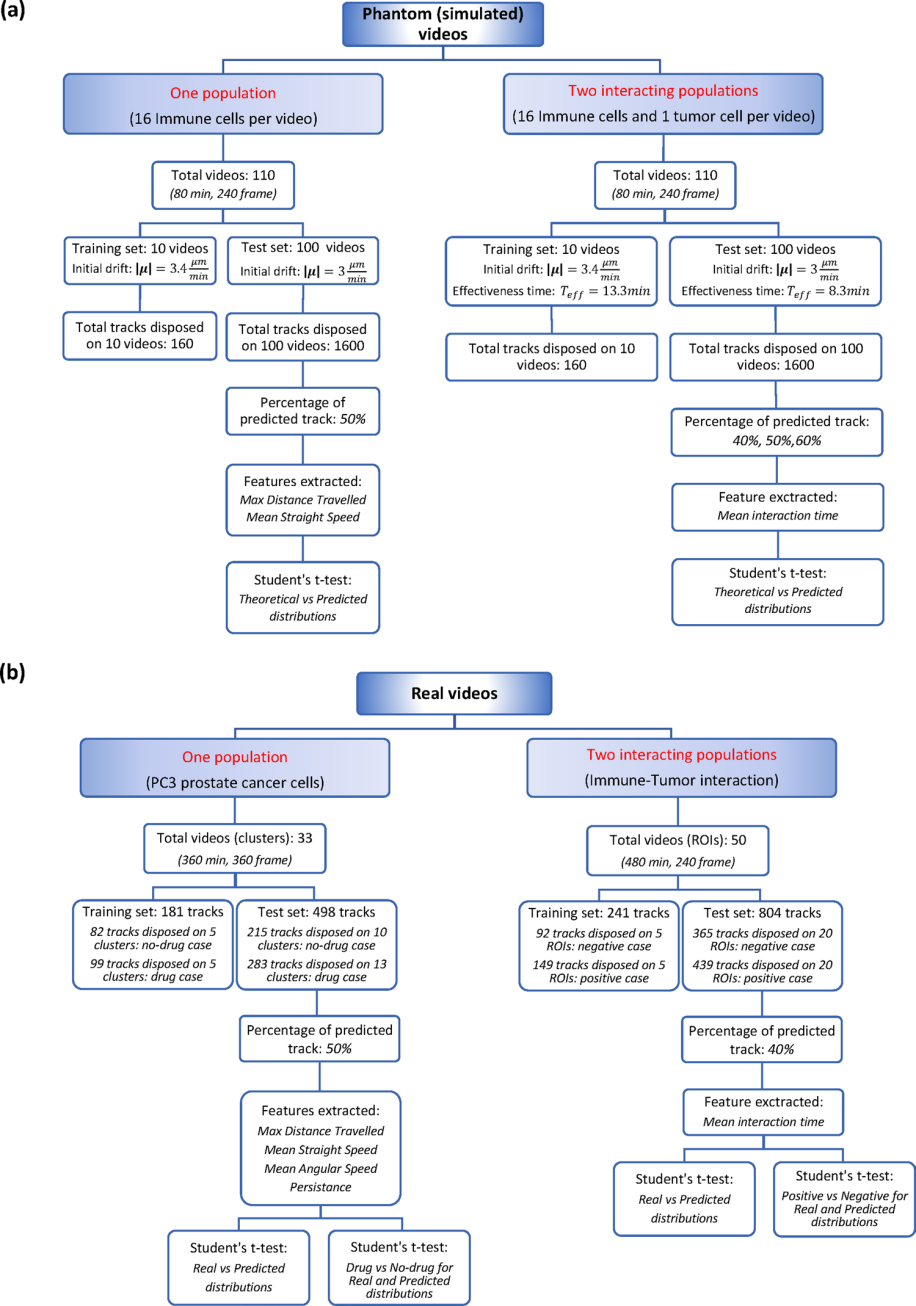
Summary of simulated and real settings. (**a**) Phantom videos. (**b**) Real videos. (**a**,**b**) The division of training and test sets, the percentages of predicted tracks, the extracted features and the performed t-tests are indicated.

In this section, we provide a sketch of the proposed approach (see Fig. 1) and a summary of the experiments (see Fig. 2) run for method validation and performance assessment.

The cells were cultured in a Petri dish or in an Organ on a chip device and time-lapse microscopy was used to acquire a video sequence of cell microenvironment (Fig. 1a). Cells were then automatically located and tracked through *Cell-Hunter* software^13,15,24^ producing a set of trajectories from the initial cell positions (Fig. 1b). The trajectories were next processed by dividing them in two parts: starting and ending slices, both counting some positions over time. The starting slices were taken in input by SGAN which iteratively predicted the socially acceptable future positions composing the ending slices, thus foreseeing the cell behavior (Fig. 1c). Finally, the ending paths of trajectories (real, i.e. detected by *Cell-Hunter*, and predicted by the network) were characterized in terms of kinematic descriptors or cell–cell interaction parameters (according to the experiment under study) for drug efficacy or tumor cell attractiveness investigations (Fig. 1d).

More in depth, we demonstrated the effectiveness of the proposed method by devising four diverse experiments, two phantom, i.e., simulated, and two real experiments. Each of them included a certain number of videos. Each video can be viewed as a “social” scene where cells of a unique population or belonging to diverse populations come in contact through interaction forces (“social forces”).

An overview of the whole experimental set-up is shown in Fig. 2. The number of analyzed videos are also specified. The two sets of phantom videos take into account one cell population, i.e., immune cells in a collective migration, and two cell populations, i.e., immune cells moving towards and then interacting with a target cancer cell, respectively (Fig. 2a). They were artificially generated by implementing stochastic migration-interaction particle models^13^. Theoretical immune trajectories obtained from the models were considered as ground truth trajectories.

On the real experiment side, a first experiment refers to one population of cells, PC-3 prostate cancer cells cultured in 2D flat Petri dishes and spontaneously disposed in clusters with different doses of the chemotherapeutic drug etoposide. Specifically, untreated (no-drug case in Fig. 2b) and treated (drug case in Fig. 2b) cells were considered. The “social” scenes (videos) of such experiments are represented by clusters (see block to the left in Fig. 2b). As a second real experiment, we investigated the tumor-immune interaction process described in Vacchelli et al*.*^25^ by analyzing microscopy images in a microdevice (OoC) of cocultured spleen cells and apoptotic fibrosarcoma cells (MCA205) treated with anthracyclines. The efficacy of immune recruitment and anticancer response was evaluated in two distinct conditions: the control/negative case where the tumor cell ligand was not present leading to a defective crosstalk and the positive case (wild-type fibrosarcoma) with the ligand involved in antitumor effective response. For this experiment, the “social” scenes (videos) consisted in Regions of Interest (ROIs) centered in tumor cells (see block to the right in Fig. 2b).

*Cell-Hunter* software was applied on all the videos of both real experiments and the resulting trajectories of prostate cancer cells and of immune cells were assumed as the ground truth trajectories, respectively (see “Automatic tracking”). For an extensive description of all the experiments, please refer to “Methods” section.

As represented in Fig. 2, the “social” scenes (videos) belonging to each of the four experiments and their related trajectories were divided in training and test sets, respectively. Trajectories of both training and test sets were split in two temporal parts in accordance with the duration of the video constituting the experiment under study. We define such parts as starting and ending paths of the trajectories, respectively. In correspondence of each experiment, a SGAN was trained: for each “social” scene in the relative training set, the network took in input the starting cell paths involved in the “social” scene and learned to predict their immediate socially acceptable future positions (see “Social predictive architecture”).

In the test phase, the trained SGANs were used to obtain a long-term prediction according to an iterative procedure (see “Iterative test”). The total percentage of prediction in each experiment is expressed in Fig. 2. In this way, the ending paths of cell trajectories were completely reconstructed by the networks. At this point, we distinguished two kinds of ending paths: the ground truth and the predicted ones. From each of them, kinematic or interaction descriptors were extracted, according to the experiment under study (see blocks with feature extracted in Fig. 2).

The goodness of prediction was quantified in two ways. The impact over the motion characterization capability was first measured using the Student’s t-test to compare the distributions of kinematic and interaction features extracted from ground-truth and the related predicted paths (Theoretical vs Predicted in Fig. 2a for phantom videos and Real vs Predicted in Fig. 2b for real videos). As second validation proof, in correspondence of the real experiments, we performed two massive comparative tests: a drug efficacy test for the real experiment with PC-3 (Drug vs No-drug) and tumor cell attractiveness test (Negative vs Positive) for the real experiment with tumor-immune interaction. Both the real distributions of descriptors (in Fig. 2b No-drug vs Drug/Negative vs Positive for real distributions) and the predicted distributions of the same descriptors (in Fig. 2b No-drug vs Drug/ Negative vs Positive for predicted distributions) were compared by means of the Student’s t-test.

As a result from the two tests, the real distributions successfully discriminated the presence of the treatment and the attractive power of the tumor cells in the presence or not of their ligand, respectively. Such significant differences were maintained when comparing the predicted distributions. This fact permits to conclude that the potential of our approach consists of saving memory storage and reducing the time for the analysis without losing the biological information carried in the experiments.

### Social approach performance on one population kind videos

#### Phantom videos

We first evaluated the SGAN-based prediction performance on 100 phantom videos (“social” scenes) with one population kind. For each video of a duration of 80 min (240 frames), the GAN social approach iteratively forecasted the positions composing the tracks of the 16 immune cell trajectories related to the last 120 frames (i.e. the last half of the video corresponding to the last 40 min), by initially taking in input the positions of the parts of the same cell trajectories lying in the first 120 frames (i.e. the first half of the video corresponding to the first 40 min). We globally tested the predictive method over 1600 cell trajectories from 100 phantom videos (Fig. 2a).

**Figure 3:**
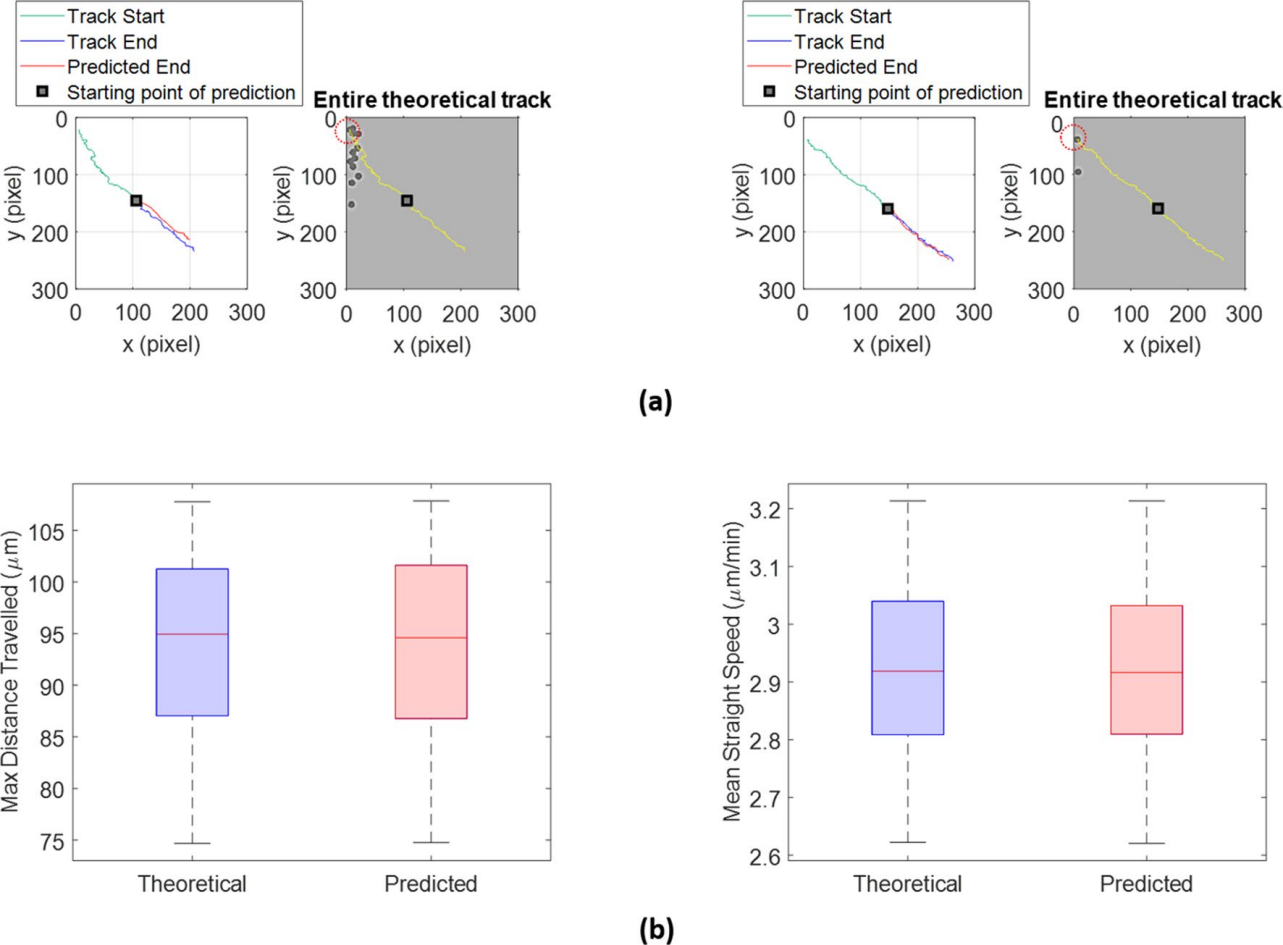
The social predictive approach effectively predicts cell trajectories of phantom videos with one population kind. (**a**) Left panels represent examples of predicted track endings (red) with the relative ground truth track endings (blue) and the ground truth track start (green). Right panels show the entire theoretical (ground truth) tracks (yellow) in phantom videos. Red dotted circles denote the cells superimposed on the tracks in yellow. (**b**) Boxplots of the max distance travelled (left) and the mean straight-line speed (right) for theoretical and predicted track endings*.* The *p* values obtained by means of the Student’s t-test are: *p* value = 0.7553 for the max distance travelled, and *p* value = 0.9191 for the mean straight speed.

Figure 3a shows two examples of prediction. In left panels, the starting part of the trajectories (green) with the predicted track endings (red) and the relative theoretical (ground truth) track endings (blue) are shown. In right panels, the entire theoretical (ground truth) cell trajectories are visualized in yellow. Cells which described such trajectories are marked with a red dotted circle.

In support of a qualitative similarity between predicted and ground-truth cell trajectories exposed in Fig. 3a, we quantified such similarity by comparing some kinematic descriptors extracted from ground truth and the relative predicted path endings (see “Statistical analysis”). In this way, we picked up descriptors over only parts of trajectories lying in the last half of all 100 phantom videos. For each extracted feature, the distributions obtained from ground truth and from predicted track slices of the overall 1600 cell trajectories were compared using the Student’s t-test. We called such distributions as Theoretical and Predicted, respectively. From boxplot comparison, in Fig. 3b, Theoretical and Predicted distributions exhibited similar means and dispersions. Indeed, a statistically significant equality between each couple of distributions (Theoretical vs Predicted) for the chosen parameters clearly appeared: *p* value = 0.7553 for the max distance travelled and *p* value = 0.9191 for the mean straight speed were obtained. In other words, the prediction globally conveyed a good estimation of parameters.

#### Real videos

Videos (“social” scenes) showing clusters of prostate cancer cells under the effect or not of chemotherapeutic drug (No-drug *vs* Drug) were analyzed. Each video had a duration of 6 h (360 frames) and the social approach iteratively predicted the cancer cell track slices lying in the last 180 frames of the all videos (i.e. the last half of the videos corresponding to 3 h), by initially taking in input the positions that formed the parts of the same cell trajectories belonging to the first 180 frames (i.e. the first half of the videos corresponding to 3 h). A total of 498 cell trajectories were involved in the test phase: 215 cells for the no-drug case and 283 cells for the drug case (Fig. 2b).

**Figure 4:**
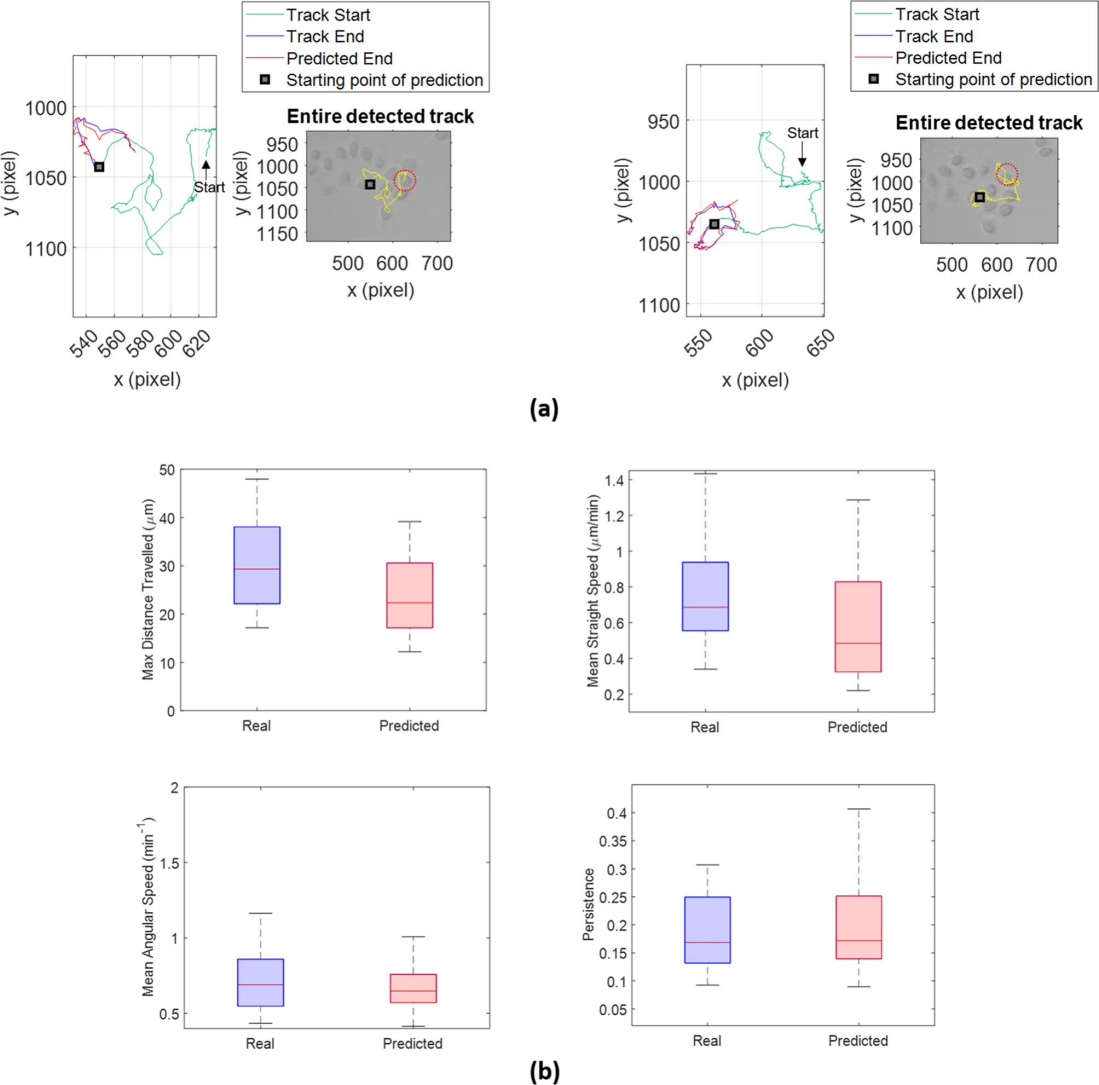
The social predictive approach effectively predicts cell trajectories of real videos with one population kind. (**a**) Left panels represent examples of predicted track endings (red) with the relative ground truth track endings (blue) and the ground truth track start (green). Right panels show the entire detected (ground truth) tracks by *Cell Hunter* (yellow). Red dotted circles denote the detected cells superimposed on the tracks in yellow. (**b**) Boxplots of the max distance travelled (upper-left), the mean straight-line speed (upper-right), the mean angular speed (lower-left) and the persistence (lower-right) for real and predicted track endings relative to the no-drug case. The *p* values obtained by means of the Student’s t-test are: *p* value = 0.5267 for max distance travelled, *p* value = 0.7356 for the main straight speed, *p* value = 0.4845 for the mean angular speed, and *p* value = 0.3540 for the persistence.

Left panels in Fig. 4a highlight examples of prediction by reporting ground truth (blue) as well as predicted path endings (red), whereas right panels show the total ground-truth trajectory, i.e. trajectory detected by means of *Cell Hunter* tool.

Following the analysis procedure, we collected some kinematic descriptors from ground truth and predicted track slices belonging to the last half of the videos, lining up real and predicted distributions. Beyond the mean straight-line speed and the max distance travelled, the persistence and the mean angular speed were extracted (see “Methods”). Here, in fact, we concerned not only to quantify the robustness of prediction in terms of total direction and speed but we needed parameters which measured the overall impact of drug on cell motility^15^. Panels of Fig. 4b depict boxplots in comparison between real and predicted distributions for each of the parameters in the control case (No-drug). Boxplots were almost totally overlapped with *p* values for the Student’s t-test higher than the cut-off 0.05: *p* value = 0.5267 for max distance travelled, *p* value = 0.7356 for the main straight speed, *p* value = 0.4845 for the mean angular speed and *p* value = 0.3540 for the persistence.

**Figure 5:**
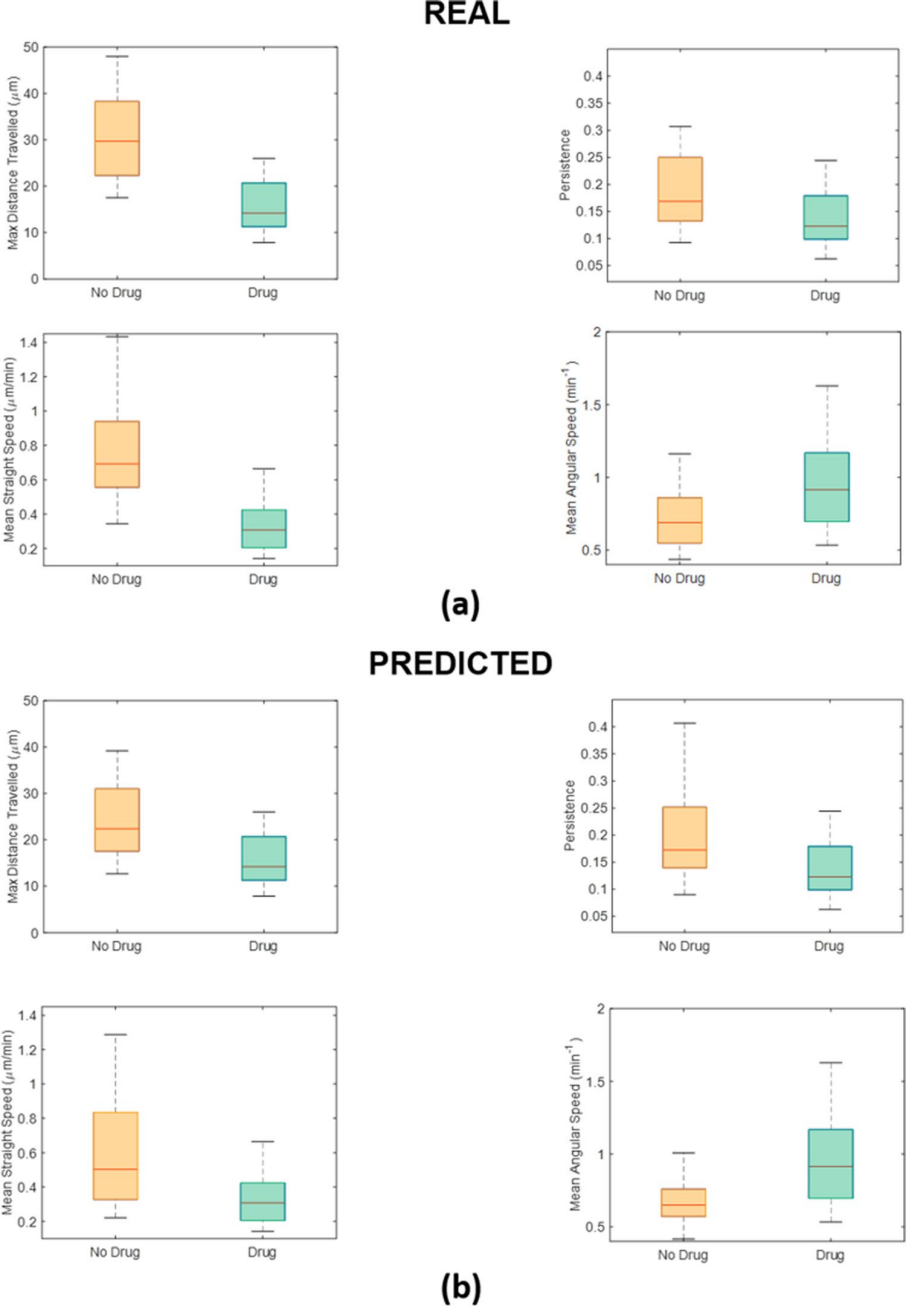
Comparison between diverse experimental conditions (no-drug *vs* drug) for real videos with one population kind. (**a**) Drug efficacy test for real distributions of motility descriptors. Boxplots of the max distance travelled (upper-left), the mean straight-line speed (lower-left), the persistence (upper-right) and the mean angular speed (lower-right) for the two experimental conditions. The *p* values obtained by means of the Student’s t-test are: *p* value = 2.17 × 10^–10^ for the max distance travelled, *p* value = 1.40 × 10^–8^ for the mean straight speed, *p* value = 1.30 × 10^–5^ for the persistence and *p* value = 3.00 × 10^–6^ for the mean angular speed. (**b**) Drug efficacy test for predicted distributions of motility descriptors. Boxplots of the max distance travelled (upper-left), the mean straight-line speed (lower-left), the persistence (upper-right), and the mean angular speed (lower-right) for the two experimental conditions. The *p* values obtained by means of the Student’s t-test are: *p* value = 2.65 × 10^–4^ for the max distance travelled, *p* value = 3.25 × 10^–5^ for the mean straight speed, *p* value = 1.57 × 10^–4^ for the persistence, and *p* value = 2.28 × 10^–4^ for the mean angular speed.

Moreover, we implemented a drug efficacy test by comparing the effect of the presence of a chemotherapeutic agent vs the control case (drug *vs* No-Drug) on cancer cell motility either for real trajectories or predicted counterparts. For each of the descriptors mentioned before, we considered comparisons of couple of distributions: No-drug vs Drug for real distributions (Fig. 5a) and No-drug vs Drug for predicted distributions (Fig. 5b). We wanted to observe if trends of distributions for the four parameters and their discriminatory power regarding the presence of drug were preserved going from real to predicted distributions. In accordance with a previous study^15^, the descriptors computed for real distributions significantly discriminated the presence of the treatment (*p* value = 2.17 × 10^–10^ for the max distance travelled, *p* value = 1.40 × 10^–8^ for the mean straight speed, *p* value = 1.30 × 10^–5^ for the persistence and *p* value = 3.00 × 10^–6^ for the mean angular speed were derived from the Students’ t-test). Drug administration globally inhibited cell motility, forcing cells to describe trajectories of a more confined motion. As consequence, cell paths became more returning with smaller displacement and speed. Indeed, the treated case recognized lower values of max distance travelled (upper-left panel in Fig. 5a), of mean straight-line speed (lower-left panel in Fig. 5a) and of persistence (upper-right panel in Fig. 5a) and higher values of mean angular speed (lower-right panel in Fig. 5a) respect to the not treated case.

As highlighted in Fig. 5b, the drug efficacy test for the predicted distributions strongly confirmed results achieved for the real ones (*p* value = 2.65 × 10^–4^ for the max distance travelled, *p* value = 3.25 × 10^–5^ for the mean straight speed, *p* value = 1.57 × 10^–4^ for the persistence and *p* value = 2.28 × 10^–4^ for the mean angular speed).

### Social approach performance on interacting tumor-immune cells videos in Organ-on-Chip experiments

#### Phantom videos

We tested the prediction capability of the social methodology on 100 phantom videos (“social” scenes) with tumor-immune interaction for a total of 1600 immune cells. As in the case of phantom videos with one population kind, we predicted trajectories of immune cell trajectories. Here the aim was to compute the mean interaction time in order to quantify the tumor-immune interaction (see “Statistical analysis”). The theoretical trajectories of tumor cells were involved in the computation of the mean interaction time for fixing the position of the tumor cell around which to define the interaction radius (see “Statistical analysis”). About prediction, the SGAN network initially received in input some positions of immune cells lying in a defined percentage of the videos and iteratively predicted the future positions up the end of the videos.

**Figure 6:**
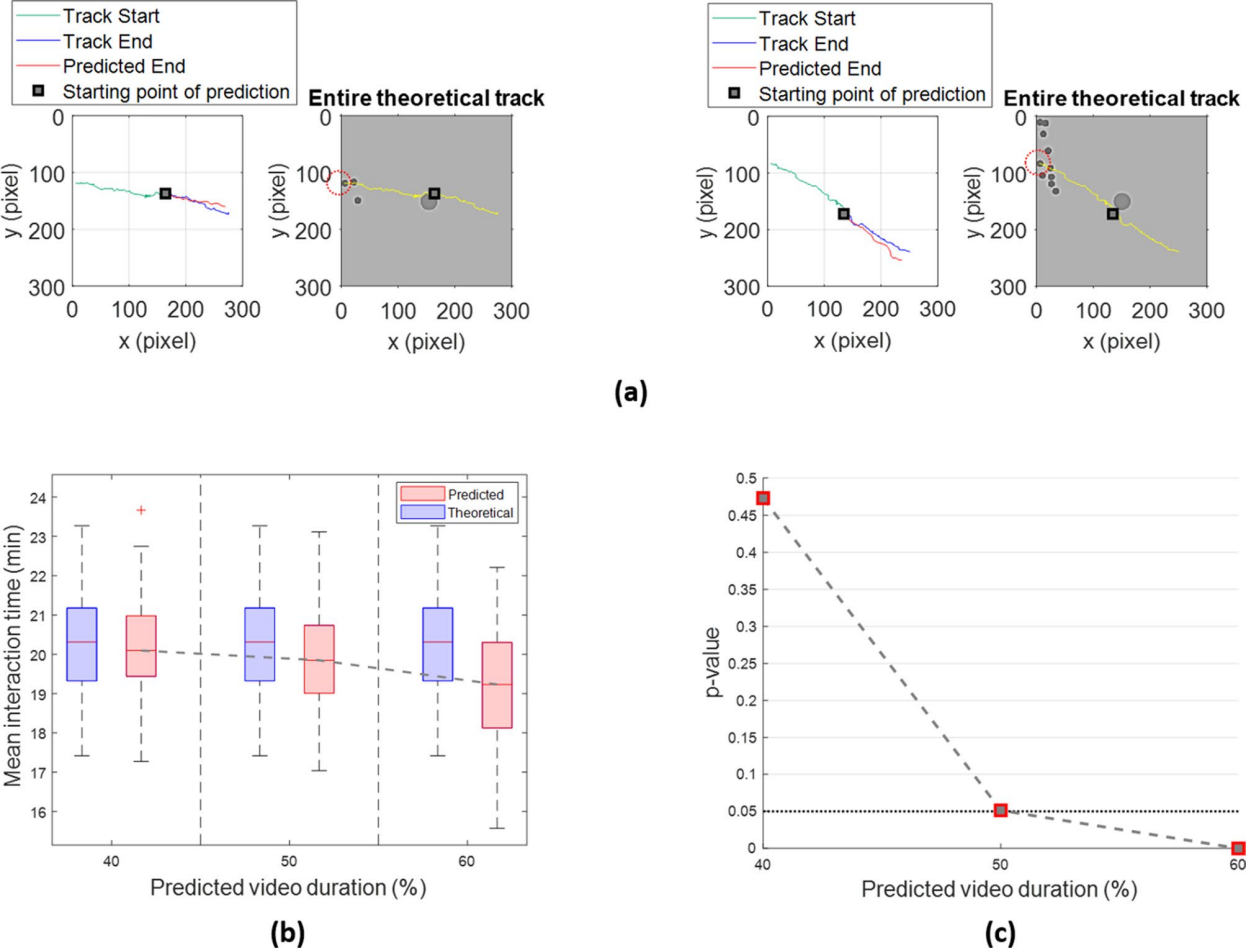
The social predictive approach effectively predicts immune cell trajectories of phantom videos with two interacting populations. (**a**) Left panels represent examples of predicted track endings (red) with the relative ground truth track endings (blue) and the ground truth track start (green). Right panels show the entire theoretical (ground truth) tracks (yellow). Red dotted circles denote the cells superimposed on the tracks in yellow. (**b**) Boxplots of the real and predicted mean interaction time at increasing predicted video duration, 40%, 50% and 60%. (**c**) Trend of the *p* values for the Student’s t-test at increasing predicted video duration, 40%, 50% and 60%.

Left panels of Fig. 6a depict two examples of predicted cell path endings lying in the last half (50%) of the videos (in blue) compared with the relative theoretical (ground truth) path endings (in red). In right panels, we can visualize in yellow the entire theoretical (ground truth) cell trajectories.

Since the use of phantom videos allowed a performance evaluation under ideal conditions, we made diverse length-based predictions in order to find a trade-off between the duration of prediction and the goodness of the estimation for the mean interaction time: we started from predicting cell path endings of the 1600 immune cells belonging to the last 40%, after to the last 50% up to the last 60% of the total video duration. As highlighted in Fig. 6b, in all three cases we compared the mean interaction time boxplots extracted from ground truth cell trajectories (Theoretical in the legend) with that obtained from trajectories with predicted endings (Predicted in the legend). The median of the predicted distributions decreased at increasing the prediction length. Figure 6c reveals how a similar trend characterized *p* values for the Student’s t-test, each one achieved from comparison between the theoretical and one of the three predicted distributions. *p* value became almost exactly the cut-off 0.05 when prediction length corresponded to the last 50% of the total video duration, until reaching a value under the cut-off for the prediction of bigger duration (*p* value = 1.21 × 10^–6^). In the latter case, even though both distributions represented the same scenario, the computed *p* value indicated a statistically significant difference between them. So, we found the most reliable result in correspondence of predicting cell path ending in the last 40% of the videos, i.e. 32 over 80 min.

#### Real videos

Tumor-immune interaction environment in two diverse experimental conditions, i.e. negative vs positive case, was exploited in order to test the social predictive strategy. The term negative refers to control videos (“social” scenes) where the tumor cell ligand was absent. The term positive indicates videos (“social” scenes) in which the tumor cell ligand was present, by leading to a more effective tumor-immune interaction and then to an increase of the mean interaction time values (see “Real experiments”). In compliance with outcomes reached for phantom videos, immune cell trajectory endings belonging to the last 40% of the total video duration were predicted, by taking in input positions of the same trajectories related to the first 60% of the total video. Since each video duration was of 240 frames, i.e. 480 min, the last 40% of the entire video corresponds to more than 3 h (192 min exactly). A total amount of 804 tracks were used for the test: 365 for the negative case and 495 for the positive case. Tumor cell trajectories were detected by *Cell Hunter* and involved in the computation of the mean interaction time only (see “Statistical analysis”).

**Figure 7:**
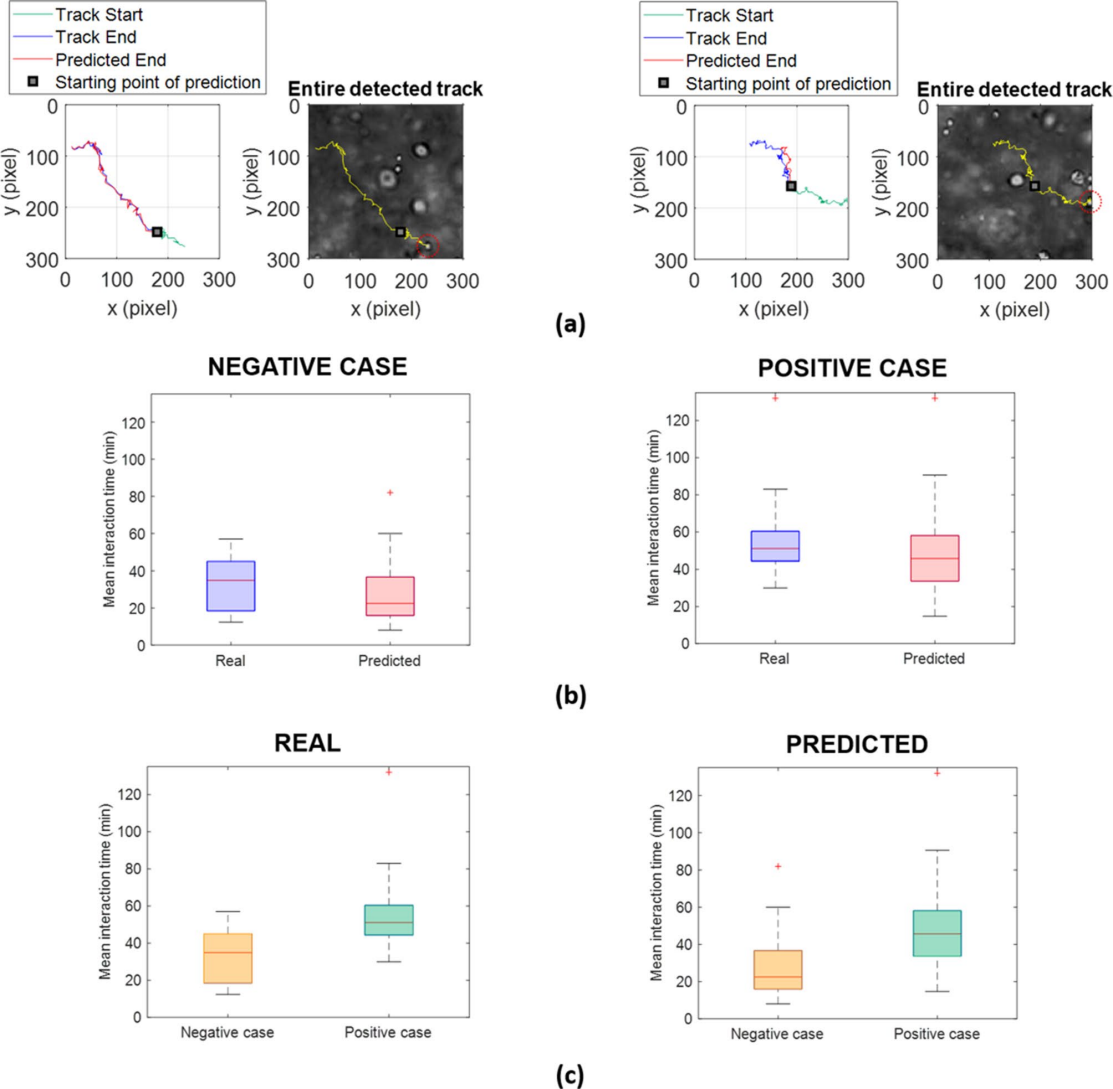
The social predictive approach effectively predicts immune cell trajectories of real videos with two interacting populations. (**a**) Left panels represent examples of predicted track endings (red) with the relative ground truth track endings (blue) and the ground truth track start (green). Right panels show the entire detected (ground truth) tracks by *Cell Hunter* (yellow). Red dotted circles denote the detected cells superimposed on the tracks in yellow. (**b**) Boxplots of the real and predicted mean interaction time in the negative case (left) and in the positive case (right). The *p* values obtained by means of the Student’s t-test are: *p* value = 0.4616 for the mean interaction time in the negative case and *p* value = 0.3381 for the mean interaction time in the positive case. (**c**) Comparison between real mean interaction time distributions for negative and positive cases (left). Comparison between predicted mean interaction time distributions of negative and positive cases (right). The *p* values obtained by means of the Student’s t-test are: *p* value = 1.43 × 10^–4^ for the mean interaction time in the real case and *p* value = 0.0087 for the mean interaction time in the predicted case.

In left panels of Fig. 7a, two examples of predicted cell path endings lying in the last 40% of the videos (in blue) compared with the relative ground truth path endings (in red) are shown. In right panels, on the real experimental background, the entire cell trajectories identified through *Cell Hunter* are drawn in yellow.

Analogously to phantom videos, we confronted the mean interaction distribution computed from tracks with predicted endings (Predicted distribution) with that from ground truth trajectories (Real distribution) in terms of *p* value for the Student t-test. Figure 7b reports boxplots of real and predicted mean interaction time in the negative (left panel) as well as positive case (right panel). In both cases, the mean interaction time did not discriminate the predicted distribution from the real one.

To exhaustively conclude the analysis, we investigated the discriminatory ability of mean interaction time between the negative and the positive case and if findings drawn for Negative *vs* Positive real distributions could be translated to the predicted counterparts. As expected from a biological point of view, the left panel of Fig. 7c points out that the absence of the tumor ligand (negative case) led to significative lower mean interaction time values as against values of the interacting descriptor in the positive case, where the presence of the tumor ligand promotes the immune response (*p* value = 1.43 × 10^–4^). For the predicted counterpart, as highlighted in the right panels of Fig. 7c, trends of distributions and the discriminatory capacity of the mean interaction time were still valid (*p* value = 0.0087).

### Comparison with competing motility prediction methods

Here we present a pipeline that uses a deep neural network to predict positions of a wide variety of cell types in a certain number of frames in movies. The strength of the proposed method may be demonstrated by showing a straightforward comparison of performance with the iterative versions of two competing predictive methods: the baseline linear kinematic prediction model^16,22^, later *Baseline model*, as well as an alternative deep learning method^16^, based on Recurrent Neural Networks (RNNs), later *RNN prediction model* (see “Competing motility prediction methods”).

Such two methods exhibit constraints of applicability in real occurrences that our model effectively overcame, ensuring its enforceability in disparate complex biological environments.

On the one hand, the assumption of ballistic motion for the Baseline model is not valid for all cell kinds. PC-3 cancer cells represent an example of a cellular population which violate this assumption because their motility is heterogeneous and could evolve as a transition among different motion kinds^26^.

On the other hand, the fixed dimension of input cell tracks constitutes the principal drawback for the RNN prediction model. Such crucial issue has already been discussed in the state of art^12^, where researchers have been guaranteed the possibility to analyze cell tracks of the same length through the introduction of ‘dummy points’, i.e. hand-made points to repeatedly add to tracks until a prefixed length equivalent to the experiment duration. However, in some scenarios, it does not make physical sense to equalize track lengths, e.g. in the proposed two interacting population scenario where immune cells constantly appear and subsequently leave the field of view in diverse time frames along with the videos. As results, cell trajectories may exhibit widely differing lengths.

Under such considerations, to ensure a more equal comparison among the three methods, we only considered movies from real experiments with one population kind (PC-3 cell line) where the lengths of all cell tracks detected by *Cell Hunter* were not equal too, but the discrepancy among lengths of the involved trajectories was drastically lower than the case previously discussed. Thus, for the *RNN prediction model,* we artificially integrated cell trajectories with ‘dummy points’ by replicating the missing points, i.e. initial and/or last positions at the beginning and/or at the end of the tracks, respectively. The other commonly used approach is zero-padding, but we preferred point repetition to avoid abrupt changes of cell position during video sequences^12^. Anyway, as we demonstrate later, the presence of such points as complement of cell trajectories dramatically affects the achieved results.

Moreover, in order to compare results obtained by the three methodologies under the same conditions, we implemented iterative versions of the abovementioned competing models on the same rationale of our social predictive method (see “Iterative test” and Fig. 9b). We employed the *Baseline model* on all the tracks of PC-3 test set by computing the mean velocity on $\tau = 9$ time steps previous the predicted position, in agreement with the optimization for $\tau$ by parameter research^16^. For the *RNN prediction model*, we adopted the training parameters described in Kimmel et al*.*^16^ by considering the same train and test sets of our model.

**Figure 8:**
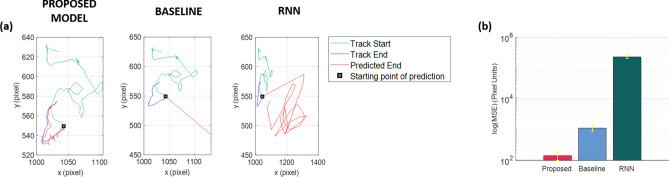
Motility prediction performance on real videos with one population kind. (**a**) Example of a predicted track end (red) by the proposed model (left), the *Baseline model* (center) and the *RNN prediction model* (right). The ground truth (real) track end and start are also highlighted in blue and green, respectively. (**b**) Mean squared error plot in log scale between ground truth and predicted track endings relative to the proposed model, the *Baseline model* and the *RNN prediction model*.

Predictions of the same track end for the three comparative models are juxtaposed in Fig. 8a, where we can observe how our method qualitatively produced a more reliable prediction. The qualitative result was confirmed in quantitative terms by computing the average mean squared error (MSE) between the predicted and the relative ground truth track slices. Figure 8b summarizes the average MSEs in log scale for all the three models. The naïve *Baseline model* reached an average MSE in pixel units of $\approx 1096$. Differently from Kimmel et al*.*^16^, the *RNN prediction model* performs worse than the *Baseline model* leading to a very higher MSE of $\approx 2.293 \times 10^{5}$ pixel units. Two factors leveraged such result: undoubtedly the introduction of dummy points but also the loss of robustness of the method in receiving in input predicted positions. Anyway, our model outperforms both comparative methods providing a significantly lower MSE of $\approx 138.6$ (pixel units).

## Discussion

The suggested predictive approach does not only recapitulate state-of-art findings on cell motility prediction but also discovers new frontiers to long-term predictions with large feasibility of applicability. The main potential of the approach is that no a priori model of motion is required and that cell trajectories of any length can be analyzed without any sizing limitations.

Motility prediction may increase the robustness and accuracy in tracking tasks considering the social planning of cells. Although sophisticated tracking algorithms have been increasingly designed to probe previously infeasible scenarios, tracking, by its nature represents a multi-object problem^27^, and therefore continues to remain a challenging issue^28^ especially in crowded environments where “social forces” among cells come into play.

As discussed in Kimmel et al*.*^16^ and Yamaguchi et al*.*^29^, motion prediction may positively impact the case of missed detections, e.g. when a cell or in general an agent can be not detected due to occlusion between multiple targets. Additionally, social forces may influence more trustworthy trajectory prediction which in turn can take advantages for tracking performances. Thus, having future information on what we observe may be essential for successful tracking.

We also underline that the major relevant aspect of our social method is the ability to predict many steps in the future reaching hours of motility prediction by taking in input not only real but also previously predicted cell positions. Such a method may be an essential building block of an envisioned potential in silico platform. The synergic contribution of time-lapse microscopy, cell motility prediction and analysis made the platform usability and reliability to have fundamental consequences for the biomedical community such as that of reducing animal experimentation and optimizing pharmacological testing from aesthetic to therapeutic purposes towards personalized medicine^30^.

Future tasks for long-term cell trajectory prediction are related to the possibility to combine prediction and acquisition in a unique pipeline. This is crucial when observing highly dynamic phenomena in which cell kinematics modes change during the experiment thus requiring the alternation of prediction and acquisition to adapt to changes.

Forward motility prediction can be also framed in a loop scenario where the parameters of the tracking and prediction algorithms are tuned according to the feedback provided by ongoing experimental results. In this way, it is possible to foresee the possibility to perform very long-term experiments (in the order of several days) enlarging the plethora of biological phenomena that can be investigated.

The effectiveness of the platform in terms of time consuming and flexibility can be also correlated to the availability of an online processing of the trajectories during cell prediction. This possibility can foresee the scenario in which memory storage is minimized since video sequences and trajectories analyzed are ongoing eliminated after decision is taken.

This facility opens new scenarios, not less relevant, related to the possibility to conduct massive analysis of collective cell trajectories with the main task to accelerate the uptake of OoC experiments and to increase the trustworthiness of software tools for immediate biological experiments exploitation.

## Methods

### Social predictive architecture

**Figure 9:**
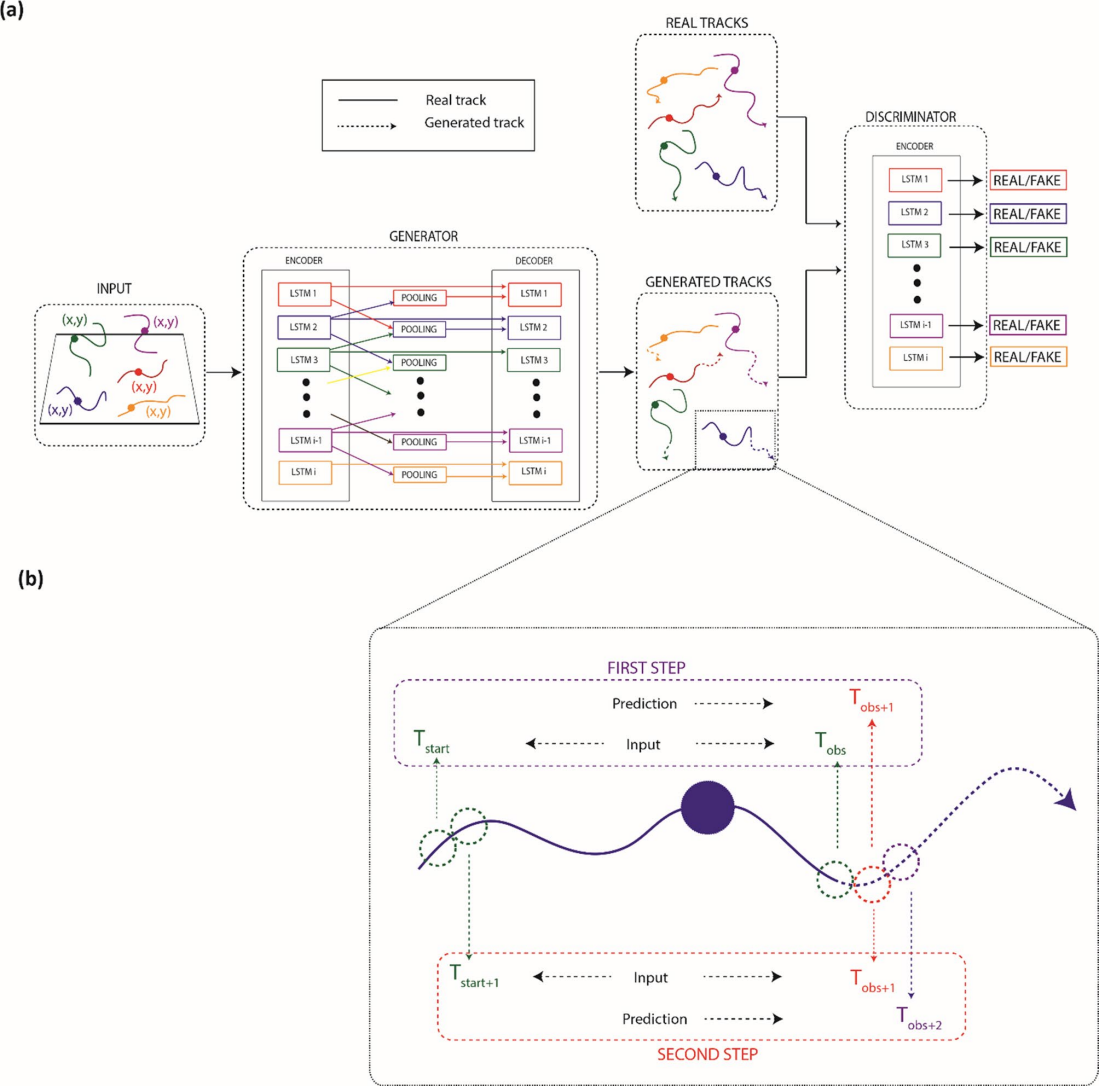
(**a**) A general framework of the social predictive algorithm. The SGAN architecture deploys the generator as an encoder-decoder, linked through a pooling module, and the discriminator as an encoder. Generator inputs the past trajectories of cells and outputs predicted trajectories exploiting the pooling module to involve social interactions among cells. The discriminator inputs both real and generated trajectories and classifies them as real or fake. The generated trajectories are composed by the real input sequence (continuous line) and the future prediction (dotted line). Real trajectories are composed by the real input sequence and the future ground-truth positions. (**b**) An overview of the two initial steps for the social predictive algorithm**.**

Our predictive framework is based on a previous design of neural network, the Social Generative Adversarial Network (SGAN), proposed in Gupta et al*.*^17^. We retained the core structure of such network for which we give an overview in Fig. 9a. It consisted of a GAN based encoder-decoder architecture integrated of a pooling layer with the goal of jointly predicting the future trajectories of all agents (cells/humans) in a “social” scene.

A Generative Adversarial Network (GAN) is a generative model characterized by two neural networks^31^, called generator and discriminator, competing in a min–max game. Essentially, the generator outputs samples taking noise as input while discriminator learns to distinguish between samples created from the generator (fake samples) and samples drawn from the input data (true samples).

From the primary GAN architecture, generator and discriminator has been widely re-modeled in literature to obtain versions of the network suitable to address the most disparate classification^32^ or prediction^33^ problems. Among them we focused on prediction of human positions^17,34^. Gupta et al*.*^17^ modelled generator as an RNN encoder-decoder and the discriminator as an RNN encoder. Generator’s encoder took as input the past trajectories of all agents individually so that learning the state of agents and encoding their history motion: a Multi-Layer Perceptron (MLP) embedded positions of each agent and then such embeddings became input of Long-Short-Term Memory (LSTM) networks as many as the number of agents in the scene. LSTMs effectively learned long-range dependencies without loss of short-time lag capabilities^35^ within agent trajectories. To sight social reasons among agents, Gupta et al*.*^17^ introduced a pooling module by combining social information from all the agents and by preventing collisions in short and far distances. To our aim, we accepted far-away cell trajectory intersections contrary to immediate collisions, so we were interested in local information about the interaction. Thus, we replaced this module in favor of the Social Pooling module proposed by Alahi et al*.*^34^, whereby LSTMs were connected each other and the hidden states of the neighbors within a certain distance were pooled. Given an agent, the identification of neighbors addressed a grid centered at the agent position under consideration. This model learned to relocate a trajectory to avert nearby agent-agent impact. Like the encoder, the decoder was composed by LSTMs, one for each agent. In this case, each LSTM predicted the future trajectory conditioned to the pooled information as well as the agent history motion (see Fig. 9a).

Finally, the last component of the network was a discriminator, modeled as a separate encoder. As shown in Fig. 9a, the discriminator received in input either real or generated trajectories and by the implementation of a MLP in the last hidden state, it classified trajectories as real or fake. Generated trajectories refer to the sequences with input trajectories (continuous line) and the future prediction (dotted line), as highlighted in the legend of Fig. 9a. The future prediction corresponds to the future ground-truth position in the real trajectories. For an in-depth discussion, please refer to Gupta et al*.* and Alahi et al.^17,34^.

### Simulated experiments mimicking cell motility

We artificially mimicked two distinct and biologically relevant motion models involving one population kind and two interacting populations, respectively.

Two atlases of 110 videos each were taken into account: the first one with one population kind, meaning a collective migration of 16 immune cells per video, and the second one with two interacting populations, where 16 immune cells per video migrated towards a tumor cell with a consequent tumor-immune interaction. The first atlas is a first-time adoption. Conversely, the latter one constitutes a data-set already generated in our previous work^13^. For 100 videos of each of the two cases, the migration was modelled with a random walk with drift, constant in modulus, $\left| {\varvec{\mu}} \right| = 3\frac{\upmu m}{{min}}$ and physical interactions among cells of the same population were modelled as repulsive potential forces. Moreover, for the 100 videos of the case of two interacting populations for which we imposed $\left| {\varvec{\mu}} \right| = 3\frac{{\upmu {\text{m}}}}{{\min}},$ the tumor-immune interaction consisted of an attractive potential force acting on immune cells within an interaction radius in proximity of the tumor cell for a priori imposed time $T_{eff} = 8.3 \,\,{\min}$, called the effectiveness time. The motion of the tumor cell was modelled as a pure random walk. Please refer to Comes et al*.*^13^ for mathematical equations. Geometrical constraints of the models were chosen by inspiring to real experiments^25^, as expressed in Comes et al*.*^13^. Immune theoretical trajectories extracted from such two sets of movies formed two distinct test sets for the predictive validation of the social method. In detail, 1600 trajectories for the test set of the se of one population kind and 1600 trajectories for the test set of the case of the two interacting populations were globally collected. Each one of the corresponding training sets of the two cases counted 160 immune theoretical trajectories extracted from 10 videos obtained by varying the drift, $\left| {\varvec{\mu}} \right| = 3.4\frac{{\upmu {\text{m}}}}{{\min}}$ for those of one population kind and the drift as well as the effectiveness time, ${ }T_{eff} = 13.3 \,\,{\min}$ for those with two interacting populations. Each video was 80 min long, i.e. it counts 240 frames (1 frame every 20 s). Theoretical tumor cell trajectories were involved in the computation of the mean interaction time (see “Statistical analysis”).

### Real experiments

Two totally diverse setting were examined: experiments with one population kind and with two interacting populations, respectively.

*One population kind* refers to PC-3 prostate cancer cells cultured in 2D Petri dishes and spontaneously arranged in clusters^36^. Two diverse experimental conditions involving not treated and treated cells with a chemotherapeutic drug (etoposide at concentration of 50 μM) were surveyed**.** We labelled the control scenario as no-drug and the treated one as drug, respectively. For the test set, we collected a total number of 498 cell tracks over 23 clusters (10 clusters counting 215 cells for no-drug and 13 clusters counting 283 cells for drug). The training set was composed by 181 cell trajectories over 10 clusters (5 clusters counting 82 cells for no-drug and 5 cluster counting 99 cells for drug). Clusters, composed up to 40 cells, were extracted from independent experiments of a duration of 6 h. One cluster corresponds to one video. Frame acquisition was conducted every minute for a total of 360 frames. Cancer cell trajectories were detected by *Cell Hunter* tool (see “Automatic tracking”).

*Two interacting populations* refer to tumor-immune interaction described in Vacchelli et al*.*^25^, where anthracycline-treated WT (wild-type) (positive case) or Anxa1^−/−^ MCA205 fibrosarcoma cells (control/negative case) were co-cultured with WT splenocytes in microfluidic devices. To evaluate the efficacy of anti-tumor immune response, two experimental conditions were considered: the control/negative case as the experiment where the tumor cell ligand was not present (Anxa1^−/−^ MCA205 fibrosarcoma cells) leading to a defective cross-talk, while the positive case as the experiment where the ligand was present (MCA205). For each experimental condition we considered 25 Regions of Interest (ROIs) centred in the tumor cells, of which 5 for training and 20 for testing. Such movies lasted 480 min, i.e. 240 frames, with acquisition of 1 frame every 2 min. Immune cell trajectories were detected by *Cell Hunter* tool (see “Automatic tracking”). We picked up 457 trajectories for the negative case (92 for training and 365 for test) and 588 trajectories for the positive case (149 for training and 439 for test), for a total amount of 1045 tracks. We were limited to analyze immune cell dynamics since tumor cells were roughly static. However, tumor cell trajectories were detected by *Cell Hunter* and used for the computation of the mean interaction time (see “Statistical analysis”).

### Automatic tracking

We applied *Cell Hunter* tracking software^13,24^ for Single Particle Tracking (STP) in movies concerning real experiments. *Cell Hunter* tool implements the segmentation method of the Circular Hough Transform (CHT)^37^, which allows to contemporarily localize cell nuclei in a given frame, by hypothesizing cells as circular-shaped objects. After frame-based localization, an upgraded version of the Munkres’ algorithm for non-square Optimal Assignment problem was used to sequentially link cell positions along the video frames, leading to the final layout of cell trajectories. Cell occlusion and overlapping problems were solved through the geometrical mechanism behind. Please refer to Comes et al*.*^13^ for a detailed explanation of the method.

### Iterative test

SGAN approach was previously applied by Gupta et al*.* to predict human trajectories up to 4.2 s in the future by observing input trajectories for 3.2 s^17^. Aware of the power of this method, we innovatively transferred the argument to predict cell trajectories in multiple experimental scenarios. Moreover, differently from Gupta et al*.*^17^, we tempted to predict long-term trajectories. In view of our purpose, we modified the method proposed by Gupta et al.^17^ in an iterative methodology for the test phase. The first two steps are highlighted in Fig. 9b.

Let be defined the input positional coordinates of the $i$th cell as $X_{i}^{t} = \left( {x_{i}^{t} , y_{i}^{t} } \right)$ from time steps $t = T_{start} , \ldots , T_{obs}$, where $T_{start}$ and $T_{obs}$ indicate the starting and the ending times of observation. At the first stage, the future predicted position for the $i$th cell was the next position assumed by the cell, $\widehat{{Y_{i}^{t} }} = \left( {\widehat{{x_{i}^{t} }}, \widehat{{y_{i}^{t} }}} \right)$, with $t = T_{obs} + 1.$ In the second iterative stage, the previously predicted position was included within the input sequence so that the input time steps become $t = T_{start} + 1, \ldots , T_{obs} + 1$ and the new future predicted position corresponded to the position at time step $t = T_{obs} + 2.$ Basically, at each iterative stage, we predicted the cell location at the subsequent time step. Then we upgraded the input sequence for the new iterative stage by including the obtained predicted position and by eliminating the first input position of the previous stage. The number of inputs, $N = T_{obs} - T_{start} ,$ remained unchanged at each iterative stage.

Our enquiry dealt very different simulated and real experiments as videos of various duration by predicting cell trajectory slices belonging to their last part. For all the experiments the starting time of observation, $T_{start}$, coincides with the first video frame. Conversely, the ending time of observation, $T_{obs}$, varies according to the experiment and represents the last time before the first prediction. For a major clarification, we give an example. Let us consider real videos with one population kind, where cells were simultaneously disposed in clusters. These videos were composed by 360 frames and we predicted cell paths in the last half of videos (from frame 181 to 360). Thus, we imposed $T_{start} = 1$ and $T_{obs} = 180$.

### Implementation details

We trained a number of networks equals to the number of the diverse experiments analyzed in this essay, i.e. two simulated (phantom) and two reals to predict the immediate socially acceptable future positions of cells on a “social” scene (video). All training procedures were performed by minimizing the objective function of the min–max game between the generator and the discriminator^17^ via Adam optimizer^38^ with a batch size of 8 for 500 epochs. The learning rate and the dimensions of the hidden state are the same of Gupta et al*.*^17^.

The implementation of our social predictive approach was performed modifying the original codes made available from Gupta et al*.*^17^ on the following site: https://github.com/agrimgupta92/sgan, which included the implementation of the Social Pooling module of Alahi et al*.*^34^.

### Statistical analysis

The analysis aimed to evaluate the predictability of our method for diverse biological experiments through the computation of kinematic or interaction descriptors on phantom as well as real videos either with one cell population kind or two diverse cell interacting populations. Specifically, for videos with one population kind, we compared distributions of each parameter collected from the predicted track endings with those extracted from the relative ground-truth track endings. The length of prediction varies experiment to experiment, as specified in the section of “Results”. For phantom videos of the latter case, max distance travelled and mean straight-line speed were calculated^39^ because they provided information about the global behavior of track slices in terms of distance and speed. Let be $$\widehat{{Y_{i}^{t} }} = \left( {\widehat{{x_{i}^{t} }}, \widehat{{y_{i}^{t} }}} \right)$$ the predicted position and $$Y_{i}^{t} = \left( {x_{i}^{t} , y_{i}^{t} } \right)$$ the ground truth position of the $$i{\text{th}}$$ cell at time $$t$$$$\in$$$$\left[ {t_{{p_{start} }} ,t_{{p_{end} }} } \right]$$ where $$t_{{p_{start} }}$$ e $$t_{{p_{end} }}$$ denote the starting and the final time of prediction, respectively. If $$Z_{i}^{t} = { }\widehat{{Y_{i}^{t} }}$$ or $$Z_{i}^{t} = Y_{i}^{t}$$ , the mean straight-line speed is defined as:

1 $$\frac{{d\left( {Z_{i}^{{t_{{p_{start} }} }} ,Z_{i}^{{t_{{p_{end} }} }} } \right)}}{{T_{i}^{p} }}$$

while the mathematical expression of max distance travelled is:

2 $${\max}\left( {d\left( {Z_{i}^{{t_{{p_{start} }} }} ,Z_{i}^{{t_{{p_{end} }} }} } \right)} \right)$$

where with $T_{i}^{p}$ the time duration of the $i{\text{th}}$ cell track from $t_{{p_{start} }}$ to $t_{{p_{end} }}$ and $d$ the Euclidean distance.

For real videos, we also extracted other two features from predicted as well as groundtruth track endings, i.e. persistence and mean angular speed, because they represented benchmarks for the evaluation of drug effect on motion inhibition^15^. Such descriptors quantify the tortuosity of cell tracks. Persistence ranges in the interval [0–1], where 1 corresponds cells moving in a perfect straight line and 0 to a very tortuous, thus confined, cell movement. Mean angular speed, instead, is proportional to path curvature, which measures how the cell path deviates from flatness. The mathematic expressions of such parameters may be found in Di Giuseppe et al*.*^15^.

Concerning the phantom videos as well as real videos showing tumor-immune interaction (two interacting populations), the predictive goodness was assessed by comparing the mean interaction time distributions^13^ obtained from ground truth trajectories with those computed from trajectories with predicted ending. Mean interaction counts the average of frames in which immune cells remains attracted to the tumor cell within an interaction radius,$R_{int} ,$ defined as the sum of the tumor and immune cell radii.

For each parameter, we quantified the difference between a couple of empirical distribution samples using the Student’s t-test. We assumed that a *p* value lower than 0.05 indicated a statistically significant difference between the two distributions.

### Competing motility prediction methods

- *Baseline model* consists of a linear regressor that estimates a cell path end by averaging the instantaneous velocity across the temporal window of $\tau$ time points preceding to the path end, where $\tau$ is optimized by means parameter research. Consult Kimmel et al*.* and Jaqaman et al*.*^16,22^ for mathematical description. The model presumes objects moving of ballistic motion or equivalently maintaining the previous observed motion direction.
- *RNN prediction model* is an architecture obtained by the conjunction of complementary functions of RNN units, such as long short-term memory (LSTM) with Convolutional Neural Network (CNN) units in order to predict motion: CNN layers may learn motility pattern as feature extractors; RNN units, instead, may learn the long-term dependencies within the input sequences.

The predictive architecture starts with input convolved by four 1D convolutional layers, then passed to an LSTM and finally convolved by other four 1D convolutional layers. All convolutional layers except the final one, which is paired with a linear activation function, use a ReLU activation. The model requires cell trajectories in input as time series disposed in a (time, x/y coordinate) matrix with a spatial dimension (time) and one channel dimension (x/y coordinate). Thus, to adopt this method, all cell trajectories should have the same length. Further details can be found in Kimmel et al*.*^16^.
